# Comparison of human bone marrow stromal cells cultured in human platelet growth factors and fetal bovine serum

**DOI:** 10.1186/s12967-018-1400-3

**Published:** 2018-03-14

**Authors:** Jiaqiang Ren, Dawn Ward, Steven Chen, Katherine Tran, Ping Jin, Marianna Sabatino, Pamela G. Robey, David F. Stroncek

**Affiliations:** 1 Cell Processing Section, Department of Transfusion Medicine, Clinical Center, National Institutes of Health, 10 Center Drive-MSC-1184, Building 10, Room 3C720, Bethesda, MD 20892-1184 USA; 2 Craniofacial and Skeletal Diseases Branch, National Institute of Dental and Craniofacial Research, National Institutes of Health, 9000 Rockville Pike, Bethesda, MD 20892 USA

**Keywords:** Bone marrow stromal cells, Mesenchymal stem cells, Fetal bovine serum, Platelet lysate, Potency

## Abstract

**Background:**

Bone marrow stromal cells (BMSCs) have classically been cultured in media supplemented with fetal bovine serum (FBS). As an alternative to FBS, pooled solvent detergent apheresis platelets, HPGF-C18, was evaluated for BMSC culture.

**Methods:**

A comparison of passage 2 BMSC growth revealed that 10% HPGF-C18 produced similar cell numbers as 20% FBS. Marrow aspirates from 5 healthy subjects were cultured for 4 passages in 10% HPGF-C18 or 20% FBS and were analyzed for proliferation, colony formation efficiency (CFE), surface marker expression, suppression of mixed lymphocyte reactions (MLRs), global gene and microRNA expression analysis. BMSC supernatant cytokine and growth factor concentrations were also compared.

**Results:**

Primary cultures of marrow aspirates in 10% HPGF-C18 and 20% FBS yielded similar numbers and CFE. After 4 passages, 10% HPGF-C18 and 20% FBS yielded similar numbers of BMSCs, surface marker expression patterns and immunosuppression effects. Gene and microRNA expression analysis revealed that BMSCs cultured under the two conditions had distinct expression profiles. Gene Set Enrichment Analysis (GSEA) revealed HPGF-C18-cultured BMSCs were enriched in metabolic processing and biosynthetic pathways, cell proliferation and cell cycle pathways, and immune response pathways. FBS-cultured BMSCs were enriched in MAPK signaling, TGF-beta signaling, cell adhesion and extracellular matrix pathways. Differently expressed microRNAs were related to the osteogenesis of BMSCs. The supernatant of HPGF-C18 BMSCs had higher levels of PEDF and TGFB1 and lower levels of IL6, VEGF, SDF1 and PLGF.

**Conclusions:**

Traditional measures, expansion, surface marker expression and inhibition of MLRs suggest that BMSC cultured in HPGF-C18 and FBS were similar, but analysis at the molecular level revealed many differences. BMSCs cultured in HPGF-C18 should be assessed in specific functional assays that reflect application-specific potency before substituting FBS with HPGF-C18.

## Background

Bone marrow stromal cells (BMSCs) are a heterogeneous population of adherent cells obtained from the marrow that have a number of potential clinical applications. Skeletal stem cells found in BMSCs produce bone and can be used for bone repair [1–3]. BMSCs also have immune modulatory properties and can induce angiogenesis and tissue repair. They are being used in early phase clinical trials to treat steroid resistant acute graft-versus-host-disease (GVHD) [4], inflammatory bowel disease [5, 6], ischemic vascular disease [7], acute lung injury [8] and traumatic brain injury [9].

BMSC were first isolated and expanded in media supplemented with fetal bovine serum (FBS) [10] and most laboratories continue to culture BMSCs in FBS. The culture of BMSCs in FBS, however, exposes the recipient of the BMSC product to potential xenogenic infection and immune reaction to bovine proteins [11–13]. In order to avoid the exposure of BMSC recipients to FBS some groups have begun to grow BMSCs in media supplemented with factors derived from human platelets. Preliminary studies have shown that when BMSCs are cultured in media supplemented with the contents of lysed platelets, they express mesenchymal stromal cells (MSC) surface markers CD73, CD90 and CD105; they differentiate in vitro into adipose tissue, chondrocytes, and osteocytes; and, in general, they proliferate faster than cells grown in FBS [14–20].

While the use of platelet lysate avoids exposure to exogenous proteins, platelet lysate is derived from human blood products and has the potential to transmit hepatitis B, hepatitis C, human immune deficiency virus and other transfusion transmitted pathogens. Many transfusion transmitted pathogens can be inactivated by various methods and platelet lysate preparations that have been treated to inactivate pathogens are available. We tested one such product, human platelet growth factor C-18 (HPGF-C18), as a source of platelet factors to support the growth of BMSCs. HPGF-C18 has been subjected to solvent-detergent treatment that inactivates lipid-enveloped viruses and to a lesser extent bacteria which reduces the risk of transmitting an infectious disease to the recipient of the BMSC products [21]. HPGF-C18 is made from a relatively large pool of outdated apheresis platelets, approximately 50 units, and it is produced following Good Manufacturing Practices (GMP) standards, both of which helps keep inter-lot variability minimal.

In this study we found that 10% HPGF-C18 produced similar numbers of BMSCs to 20% FBS in a preliminary experiment using passage 2 BMSCs. We then compared BMSCs manufactured from marrow aspirates with media supplemented with 10% HGPF-C18 or 20% FBS using the same methods that our laboratory has used to produce clinical grade BMSCs. We compared the proliferation, surface marker expression, immunosuppression effects of BMSCs and measured the concentrations of cytokines and growth factors in the supernatant. We also compared the global profiles of the BMSCs cultured by HPGF-C18 and FBS using gene expression and microRNA expression analysis.

## Methods

### Study design

The study made use of human BMSCs collected from healthy subjects. In order to determine the best concentration of HPGF-C18 for BMSCs growth passage 2 BMSCs were cultured in various concentrations of HPGF-C18 and compared to BMSCs cultured in 20% FBS, the concentration that is used in our laboratory for the production of BMSCs following GMPs [22]. After the best concentration of HPGF-C18 was identified, bone marrow aspirates were obtained from 5 healthy subjects and BMSCs were isolated and cultured for 4 passages, the cellular expansion, surface markers, and immunosuppressive activities were compared between HPGF-C18 cultured BMSCs and 20% FBS cultured BMSCs. We also measured a variety of cytokines and growth factors in the supernatant and compared the gene expression profiles and microRNA profiles of the BMSCs cultured under both conditions. These studies were approved by an NHLBI committee on the use of human subjects in research.

### HPGF-C18

HPGF-C18 was prepared from 52 outdated units of apheresis platelets each of which contained approximately 4 × 10^11^ platelets (GwoWei Technology Co, Ltd., Taipei, Taiwan). The pooled platelets were solvent/detergent (S/D)-treated (1% tri-*n*-butyl phosphate and 1% Triton X-45), extracted with oil, purified by C18 hydrophobic interaction chromatography and sterile filtered as previously described [23].

### Determination of the optimal HPGF-C18 concentration for BMSC growth

Cryopreserved passage 2 BMSCs were cultured to determine the best concentration of HPGF-C18 for BMSC growth. These passage 2 BMSCs were isolated from marrow aspirates of 3 healthy subjects using media supplemented in 20% FBS and were cryopreserved in 5% DMSO and 6% HES and stored in the vapor phase of liquid nitrogen. The passage 2 BMSCs were thawed, washed and suspended in alpha MEM with 2 mM glutamine (Lonza, Walkersville, MD), supplemented with 10 µg/mL Gentamicin and 20% lot-selected FBS (Hyclone, Thermo Fischer Scientific, Waltham, MA) or 5, 10, 15 and 20% HPGF-C18 and were seeded on T75 flasks at a density of 3000 cells/cm^2^. The cells were cultured and harvested with 5 mL TrypLE Express (Invitrogen, Life Technologies) when they reached 70–80% confluence and were re-seeded on T75 flasks at a density of 3000 cells/cm^2^ for expansion. Passage 4 cells were harvested and surface marker of passage 4 BMSCs was compared. The number of BMSCs at passage 3 and 4 was manually counted and the population doubling (PD) for each passage was calculated.

### Culture of BMSCs from marrow aspirates

The marrow aspiration and BMSC culture were performed according to standard operating procedures (SOP) established in our laboratory [22]. After obtaining informed consent, marrow was collected from the posterior iliac crest of 5 healthy donors. A total of 5–10 mL of marrow was collected in Bone Marrow Prep Syringes (Pharmacy Department, NIH, Bethesda, MD) and then washed with 2.5 × volume of HBSS (Lonza, Walkersville, MD). A single cell suspension was made with BMSC culture media (BMSC CM) [alpha MEM with 2 mM glutamine (Lonza), supplemented with 20% lot-selected FBS (Hyclone, Thermo Fischer Scientific, Waltham, MA) or 10% HPGF-C18 and 10 µg/mL gentamicin] and was plated at a density of 2 × 10^5^/cm^2^ in T-75 flasks (Corning Life Sciences, Corning, NY) and incubated at 37 °C in 5% CO_2_. Non-adherent cells were removed after 24 h; the media was changed every 3 days until the colonies reached 70–80% confluence.

The primary BMSCs were washed with 10 mL HBSS twice and lifted with 5 mL TrypLE Express (Invitrogen, Life Technologies, Grand Island, NY), the cells were then centrifuged at 406×*g* for 10 min and the cell number was counted. The cells harvested at this stage were designated as Passage 1. The BMSCs were then seeded on plastic surface at a density of 3000 cells/cm^2^, cultured and harvested as described above when they reached 70–80% confluence.

Passage 4 cells were harvested for evaluation of surface marker expression, suppression on the proliferation of mixed lymphocytes, global gene expression profiling, and microRNA expression analysis. Cytokine and growth factor levels were measured in the supernatant of passage 4 BMSCs. The number of BMSCs at passage 3 and 4 was manually counted and the population doublings (PDs) for each passage was calculated. Cumulative PDs were calculated in relation to the number of cells at the first passage.

### Primary colony-forming efficiency (CFE) enumeration

Bone marrow aspirates were diluted in culture media with 20% FBS or 10% HPGF respectively, and then plated at a density of 1 × 10^5^ per T25 flask and cultured for 13 days without changing culture medium. The colonies were fixed with methanol for 30 min and stained with saturated methyl-violet water solution for 20 min. Colonies were observed under low magnitude light microscope field (25×). Colonies containing 50 or more cells of fibroblastic morphology were counted, and CFE (number of BMSC colonies/plating nucleated cells) was calculated.

### Surface marker expression

BMSC surface markers were analyzed by flow cytometry. The cells were incubated with antibodies CD90-FITC, CD73-PE, CD146-PE, CD106-APC, CD45-FITC, CD14-PE, CD19-FITC, CD34-APC, HLA-DR-APC (BD Bioscience, San Diego, CA), CD11b-FITC and CD105-APC (eBioscience, San Diego, CA) for 20 min at 4 °C, washed on Lyse Wash Assistant (LWA, BD Bioscience) and acquired 30,000 events on a FACSCanto (BD Bioscience). The data were analyzed using FACSDiva 6.0 software (BD Bioscience).

### Cytokine and growth factor analysis of BMSC culture supernatant

The cytokine and growth factor concentrations in BMSC culture supernatant were evaluated using SearchLight Protein Array Analysis (Aushon Biosystems, Billerica, MA). Culture supernatant was collected, centrifuged for 10 min at 1400 rpm to remove cell debris and then stored at − 80 °C. The supernatants of BMSC from 5 healthy donors were evaluated for IL4, IL6, IL8, IL10, KGF, LIF, PEDF, TGFB1, FGF2, HGF, PDGFBB, VEGF, SDF1, PLGF, ANG2, and Endoglin.

### Mixed lymphocyte reaction (MLR)

The immunosuppressive properties of BMSCs were compared using MLR assay (SAIC-Frederic, Frederic, MD). Ficoll-separated peripheral blood mononuclear cells were plated in 96-well plates at 1 × 10^5^ responders per well. Responders were co-cultured with 2500 cGy irradiated stimulator peripheral blood mononuclear cells at a concentration of 1 × 10^5^ cells per well. BMSCs cultured by either 10% HPGF-C18 or 20% FBS were added at concentrations of 1 × 10^4^ and 4 × 10^4^ cells/well. Culture plates were incubated for 6 days in a humidified 5% CO_2_ incubator at 37 °C. On the day of harvest, 0.5 μ Ci of ^3^H-thymidine (^3^H-TdR) was added to each well for 4 h with lymphocyte proliferation measured using a liquid scintillation counter. The effect of BMSCs on MLR was calculated as the percentage of the suppression compared with the proliferative response of the positive control without BMSC, where the positive control was set to 0% suppression. The experiments were performed three times for each variable described.

### Microarray gene expression analysis

Total RNA was extracted using miRNeasy Mini Kit (Qiagen, Hilden, Germany) and assessed using Nano Drop 2000 (Thermo Scientific, Wilmington, DE). Microarray expression experiments were performed on 4 × 44 K Whole Human Genome Microarray (Agilent technologies, Santa Clara, CA, USA) according to our protocols [24]. Generally, 0.5 µg of BMSC RNA was labeled with Cyanine 5-CTP and Universal Human Reference RNA (Stratagene, Santa Clara, CA, USA) was labeled with Cyanine 3-CTP using a Quick Amp Labeling kit (Agilent). After purification, 825 ng of labeled cRNA from BMSC and reference RNA was pooled, fragmented and then hybridized on 4 × 44 K microarrays for 17 h at 65 °C. Images of the arrays were acquired using a microarray scanner G2505B (Agilent technologies) and image analysis was performed using feature extraction software version 9.5 (Agilent Technologies). The Agilent GE2-v5_95 protocol was applied using default settings.

### RT-qPCR analysis of gene expression

For verification of the gene expression profiling results, RT-qPCR gene expression analysis was performed using custom made PCR arrays (Qiagen). The data analysis was conducted using the ΔΔCt method. HPRT1 was used as housekeeping gene and its Ct values were used to normalize the data. The normalized ΔCt for each gene of interest (GOI) was calculated by deducting the averaged Ct of HPRT1 from the Ct of each GOI: ΔCt = (Ct^GOI^ − Ct^HPRT1^). The ΔΔCt for each GOI was calculated by deducting the average ΔCt of GOI in the HPGF group from the ΔCt of each GOI in the FBS group: ΔΔCt = average ΔCt (HPGF group)—average ΔCt (FBS group). The fold-change of each GOI in HPGF group compared to the FBS group was calculated as: fold-change = 2^(−ΔΔCt)^.

### MicroRNA expression analysis

The expression of MicroRNAs was measured by using the Human miRNome miScript miRNA PCR Array (Qiagen) following the manufacturer’s instructions. In brief, 1 µg of RNA was reverse-transcribed with miScript reverse transcription (RT) master mix for 60 min at 37 °C, then heated at 95 °C for 5 min and diluted with water. The diluted RT product was loaded into miRNA PCR Array for amplification using the following parameters, 95 °C for 1 min, then 40 cycles of amplification: 94 °C for 15 s, 55 °C for 30 s, 70 °C for 30 s, followed by the dissociation curve stage. The data analysis was conducted using ΔΔCt method. RNU6-2 was used to normalize the data. The normalized ΔCt for each miRNA of interest (MOI) was calculated by deducting the averaged Ct of RNU6-2 from the Ct of each MOI: ΔCt = (Ct^MOI^ − Ct^RNU6-2^). The ΔΔCt for each MOI was calculated by deducting the average ΔCt of MOI in the HPGF group from the ΔCt of each MOI in the FBS group: ΔΔCt = average ΔCt (HPGF group)—average ΔCt (FBS group). The fold-change of each MOI compared to the FBS group was calculated as: fold-change = 2^(−ΔΔCt)^.

### Data processing and statistical analyses

Global gene expression analysis was performed according to a standard procedure. Raw data was uploaded into mAdb database (http://madb.nci.nih.gov/) and then imported into BRB-ArrayTools [25] (http://linus.nci.nih.gov/BRB-ArrayTools.html). Tests for differences between HPGF-C18 and FBS were conducted for individual genes using paired two-sided t tests, considering P values of < 0.001 as significant. In a class prediction model, genes significantly different between the classes (HPGF-C18 vs FBS) at 0.001 significance level were used for class prediction, and the leave-one-out cross-validation method was used to compute misclassification rate. The Benjamini and Hochberg method was used to estimate the false discovery rate. Gene set enrichment analysis (GSEA) was conducted by following the instructions of Broad Institute [26] (http://www.broadinstitute.org/gsea/index.jsp).

## Results

### Optimization of HPGF-C18 concentration

**Fig. 1:**
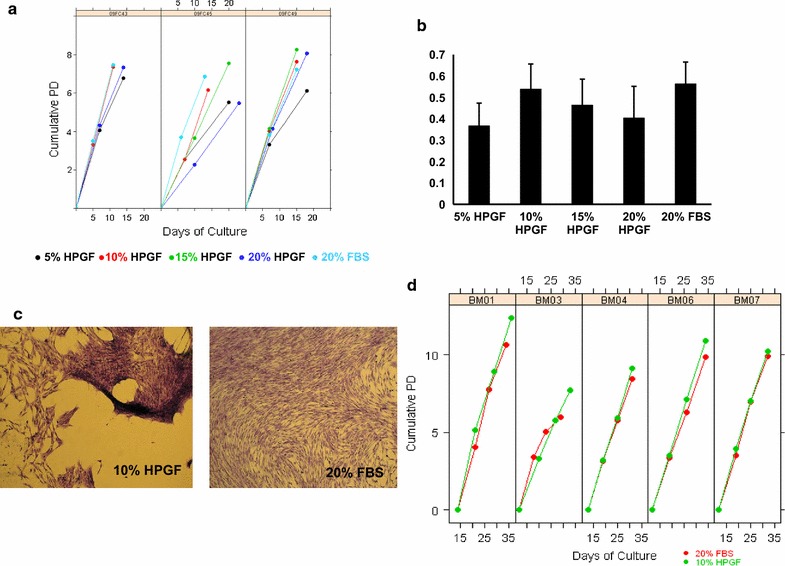
Proliferation of BMSCs in HPGF-C18 and FBS. Cryopreserved passage 2 BMSCs from 3 healthy subjects were thawed and cultured for 2 additional passages in αMEM supplemented with 5, 10, 15 and 20% HPGF-C18 and 20% FBS. The cumulative population doublings (PDs) at passages 3 and 4 are shown in **a**. The mean (± 1 SD) cumulative PDs normalized by total number of days in culture at passage 4 for each culture condition is show in **b**. Primary cultures of marrow aspirates cultured in αMEM supplemented with 10% HPGF-C18 showed irregular spacing and piling up of BMSCs while primary cultures of the same marrow aspirated cultured in 20% FBS formed more uniform monolayers (**c**). BMSCs from marrow aspirates of 5 healthy subjects were cultured for 4 passages in 10% HPGF-C18 and 20% FBS. The cumulative population doubling for BMSCs grown in 10% HPGF-C18 are shown in green and of 20% FBS in red. Each panel represents BMSCs isolated and expanded from one healthy subject. Each circle represents one BMSC passage (**d**)

In order to determine the optimum concentration of HPGF-C18 to support the growth of BMSCs, cryopreserved passage 2 BMSCs prepared from 3 healthy subjects were thawed and cultured for 2 additional passages in αMEM supplemented with 5, 10, 15 and 20% HPGF-C18 and 20% FBS. For all 3 donors, BMSCs cultured in 10% HPGF-C18 exhibited similar growth rate to those cultured in 20% FBS, and faster than those cultured in 5, 15 and 20% HPGF, except that 15% HPGF-C18 was more supportive for BMSC growth from donor 09FC49 (Fig. 1a). In summary, the proliferative rate of BMSCs as measured by populations doublings (PD) per day was greatest in BMSCs grown with 10% HPGF-C18, which was similar to that of BMSCs cultured in 20% FBS (Fig. 1b).

The proportion of BMSCs expressing CD73, CD90, CD105, CD146 and CD166 was over 90% and similar for all culture conditions, while the expression of CD106 was variable. When the mean fluorescence intensity (MFI) was compared, CD73, CD90, CD105, CD146 and CD166 had similar expression levels in BMSCs cultured in HPGF-C18 and 20% FBS. The expression of CD105 was slightly brighter in BMSCs cultured in 20% FBS than those cultured in HPGF-C18; and that of CD106 was slightly dimmer in 20% FBS cultured BMSCs (data not shown). Since BMSCs cultured in media supplemented with 10% HPGF-C18 were most similar to those cultured in 20% FBS, all further studies were performed with media supplemented with 10% HPGF-C18.

### Comparison of BMSCs Cultured in 20% FBS and 10% HPGF-C18

#### Primary BMSC Cultures

Marrow aspirates were collected from 5 healthy subjects and BMSCs were cultured for 4 passages in αMEM supplemented with 10% HPGF-C18 or 20% FBS. The primary BMSCs cultured with 10% HPGF-C18 and 20% FBS were harvested on the same day, ranging from day 12–14. Compared with BMSCs cultured in 20% FBS, the number of BMSCs harvested from 10% HPGF-C18 was similar from one subject, less from 2 subjects, and greater from 2 subjects (Table 1). It was noted that the morphology of the colonies grown in the two media supplements differed slightly. BMSCs cultured with 20% FBS formed uniform monolayers, while some colonies cultured with 10% HPGF-C18 were uneven and clumped (Fig. 1c). The media with HPGF-C18 was found to contain particles and the BMSCs appeared to clump around the particles. For the culture of the last two marrow aspirates, BM06 and BM07, the particles were allowed to settle and only the particle-reduced supernatant was used. This resulted in more uniform cell growth, but some clumping still occurred. However, this difference disappeared with cell passaging. The CFE was also evaluated for each aspirate in media supplemented with 10% HPGF-C18 and 20% FBS, and it was similar for the two media supplements for all 5 aspirates (Table 1).

**Table 1:** Comparison of primary BMSCs cultured with 10% HPGF-C18 and 20% FBS from marrow aspirates of 5 healthy subjects

	Media supplement	Number of nucleated cell from marrow	Total number of BMSCs harvested	Days in culture	CFE per 1 × 10^5^ cells
BM01	10% HPGF-C18	10.0 × 10^6^	1.20 × 10^5^	14	2
	20% FBS	10.0 × 10^6^	1.14 × 10^6^	14	1
BM03	10% HPGF-C18	6.10 × 10^6^	3.00 × 10^5^	12	4^a^
	20% FBS	6.10 × 10^6^	4.20 × 10^5^	12	3
BM04	10% HPGF-C18	55.2 × 10^6^	7.8 × 10^5^	13	5^a^
	20% FBS	55.2 × 10^6^	5.94 × 10^6^	13	4
BM06^b^	10% HPGF-C18	5.8 × 10^6^	2.2 × 10^6^	12	9^a^
	20% FBS	5.8 × 10^6^	3.78 × 10^6^	12	11
BM07^b^	10% HPGF-C18	21.7 × 10^6^	2.4 × 10^6^	12	30^a^
	20% FBS	21.7 × 10^6^	1.14 × 10^6^	12	25

^a^Colonies with atypical features

^b^Particles were allowed to settle for the preparation of HPGF-C18

#### BMSC Expansion from Passages 2 through 4

The proliferation rate of BMSCs cultured in 10% HPGF was similar to those cultured in 20% FBS but the accumulative population doublings was greater in media supplemented with 10% HPGF-C18 (Fig. 1d). The expression of stromal cell and hematopoietic cell surface markers were similar on passage 4 BMSCs cultured in 10% HPGF-C18 and 20% FBS; over 90% of cells expressed CD73, CD90, CD105, CD146 and CD166, while the expression of CD11b, CD14, CD19, CD34, CD45 and HLA-DR was almost undetectable in all BMSC samples. The expression of CD106 was higher in BMSCs cultured with 10% HPGF-C18 than those cultured with 20% FBS (Table 2).

**Table 2:** The expression of surface markers by flow cytometry

Donor	Supplement	CD90	CD73	CD105	CD146	CD166	CD106	CD11b	CD34	CD45	CD14	HLA-DR	CD19
BM01	10% HPGF	99.5	99.2	99.4	99.1	99.6	14.4	0.6	2.1	0.9	0.9	0.9	0.6
	20% FBS	99.9	99.9	99.9	99.9	99.6	12.3	0.8	1.4	0.9	0.3	0.6	0.7
BM03	10% HPGF	97.6	97.2	97	92.1	97.3	58.8	1	4	1	0.4	1.2	0.7
	20% FBS	99	99	98.9	98.5	98.9	18.3	1.1	1.3	1.1	0.3	1.3	1.3
BM04	10% HPGF	99.3	99.2	99	98.6	99.1	49.5	1	5.7	1.2	1	1.2	0.9
	20% FBS	99.9	99.9	99.9	99.8	100	26.1	1.1	9.6	0.8	1	15.6	0.7
BM06	10% HPGF	99.5	99.4	99.3	93.8	99.5	32.6	1.5	3.7	1.2	1.4	1.6	1.2
	20% FBS	100	100	100	99.4	100	8.5	1.4	2.9	1.2	1	1.2	1.5
BM07	10% HPGF	99.3	99.2	99.3	98.8	99.3	47.5	1.2	2.1	1.3	0.9	1	0.8
	20% FBS	98.7	98.8	98.8	99	99.3	31.1	1.2	3.6	1	0.1	1.1	1.3

### Suppression of MLRs by BMSCs

**Fig. 2:**
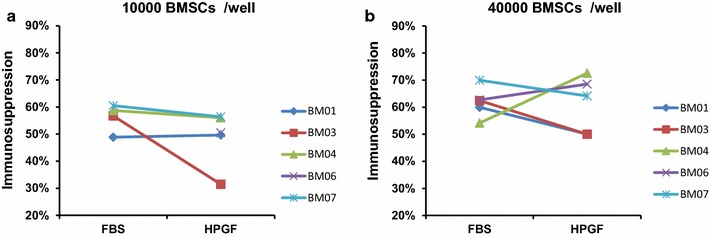
Inhibition of mixed lymphocyte reactions by BMSCs cultured with 10% HPGF-C18 and 20% FBS on the proliferation of mixed lymphocytes. BMSC suppression of mixed lymphocyte reactions was measured by H^3^-thymdine incorporation methods; the percent suppression was calculated by normalizing the values to the mixed lymphocyte reaction without BMSCs. Two doses of BMSCs were evaluated, 1 × 10^4^ BMSCs per well (**a**, data for BM06 is missing due to technical issues) and 4 × 10^4^ per well (**b**). There was no significant difference on their suppressive activity

To evaluate the immunosuppressive activity of BMSCs cultured with the 2 media supplements, their ability to suppress MLRs was evaluated using two cell doses; 10,000 BMSCs per well and 40,000 BMSCs per well with BMSC: lymphocyte ratios of 1:10 and 1:2.5 respectively. At the 10,000 BMSCs per well dose level (BMSC: lymphocyte ratio is 1:10) BMSCs cultured in HPGF-C18 displayed lower immunosuppression activity than those cultured in FBS, but the difference is not statistically significant (FBS vs HPGF, 0.56 vs 0.49, p = 0.23). At the 40,000 BMSCs per well dose level (BMSC: lymphocyte ratio is 1:2.5) BMSCs cultured in HPGF-C18 displayed similar immunosuppression activity to those cultured in FBS (FBS vs HPGF, 0.62 vs 0.61, p = 0.90) (Fig. 2).

### Concentrations of Cytokines and Growth Factors in the Culture Supernatant

**Fig. 3:**
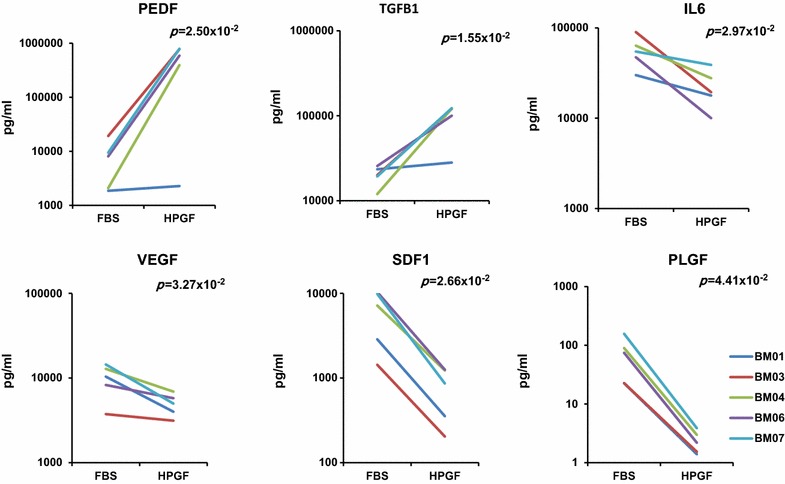
Concentration of proteins in BMSC culture supernatants. The supernatants of BMSCs cultured in 10% HPGF-C18 or 20% FBS were collected and the concentrations of proteins were measured by SearchLight. The p-values were calculated by paired t-test

The concentrations of several cytokines and growth factors were measured in BMSC culture supernatants. The specific proteins that were evaluated were those related to BMSC immunosuppressive functions and other beneficial effects of BMSCs. Among these factors IL4, IL10, bFGF and ANG2 were not detected in either HPGF-C18 or FBS BMSC supernatants (data not shown). The levels of PEDF and TGFB1 were significantly increased (p < 0.05) in the supernatant of HPGF-C18-cultured BMSCs compared to FBS-cultured BMSCs, while IL6, VEGF, SDF1 and PLGF were significantly decreased (p < 0.05) (Fig. 3). The levels of other factors did not differ significantly including IL8 (FBS vs HPGF; 300.69 vs 216.54 pg/ml, p = 0.21); KGF (FBS vs HPGF; 124.66 vs 25.77 pg/ml, p = 0.11), LIF (FBS vs HPGF; 92.80 vs 100.34 pg/ml, p = 0.77), HGF (FBS vs HPGF; 49.75 vs 38.97 pg/ml, p = 0.50). Interestingly, endoglin (CD105), which is a BMSC surface marker, was detected in the supernatant from both culture conditions, and it was significantly increased in the supernatant of HPGF-C18-cultured BMSCs (FBS vs HPGF; 544.97 vs 2497.79 pg/ml, p = 0.0028).

### Global Gene Expression Analysis

**Fig. 4:**
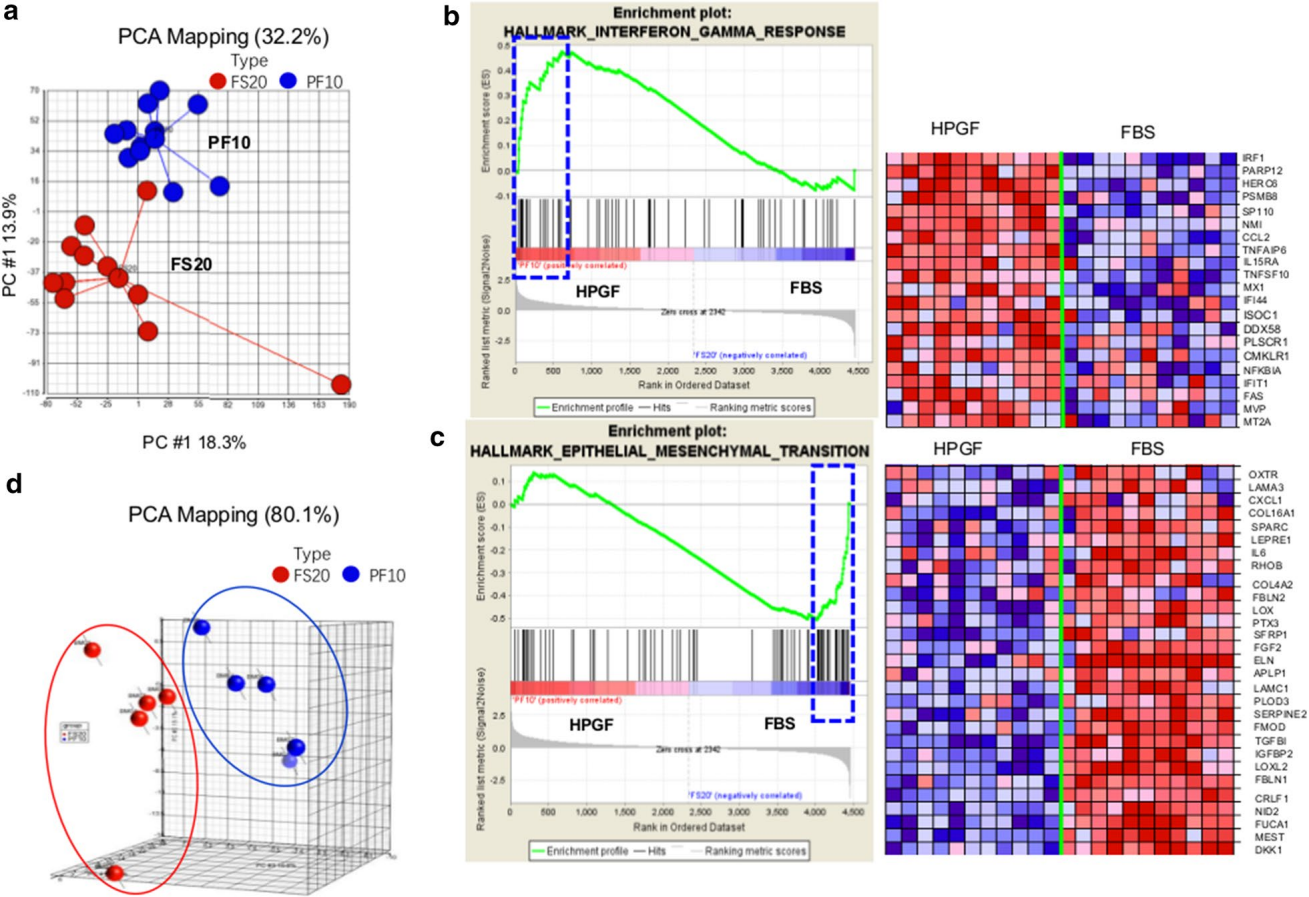
Global transcriptome and microRNA analysis of BMSCs cultured in 10% HPGF-C18 and 20% FBS. BMSCs were prepared from marrow aspirates of 5 healthy subjects and were cultured for 4 passages in 10% HPGF-C18 or 20% FBS. Passage 3 and 4 BMSCs from all 5 subjects were analyzed by global gene expression analysis. The results of the principal component analysis (PCA) analysis of the gene expression data are shown, the blue circles represent BMSCs cultured in 10% HPGF-C18 and the red circles BMSCs cultured in 20% FBS (**a**). Microarray data were analyzed using Gene Set Enrichment Analysis (GSEA) software to identify functionally related groups of genes (gene sets) with statistically significant enrichment. The enrichment plot for interferon gamma response and the 21 genes on the leading edge that are positively correlated with 10% HPGF-C18 (indicated by the blue rectangle) are shown in **b**. The enrichment plot for epithelial–mesenchymal transition and the 29 genes on the leading edge that are negatively correlated with HPGF-C18 culture (as indicated by the blue rectangle) were shown in **c**. In both **b**, **c** the plot on the left shows the distribution of genes in the set that are positively and negatively correlated with the HPGF-C18 phenotype. The plot on the right shows the relative gene expression (red = high, blue = low) for each gene for the indicated samples. Passage 4 BMCSs were also analyzed by microRNA PCR array, the PCA results on the microRNAs were shown, the blue circles represent BMSCs cultured in 10% HPGF-C18 and the red circles BMSCs cultured in 20% FBS (**d**)

Passage 3 and 4 BMSCs were analyzed by global gene expression analysis. Principal Component Analysis (PCA) revealed that the BMSCs cultured with 10% HPGF-C18 clustered separately from those cultured with 20% FBS (Fig. 4a) indicating that BMSCs cultured in the two media supplements represent two different cell types. We also used 6 class prediction methods to compare BMSCs cultured with 2 media supplements. All class prediction methods including Compound Covariate Predictor, Diagonal Linear Discriminant Analysis, 1-Nearest Neighbor, 3-Nearest Neighbors, Nearest Centroid, and Support Vector Machines separated the BMSCs cultured with 10% HPGF-C18 and 20% FBS with 100% accuracy providing further evidence that BMSCs cultured in the two media supplements represent two different cell types.

We ran Gene Set Enrichment Analysis (GSEA) for functional annotation of the difference between HPGF-C18 and FBS cultured BMSCs. We found that distinct gene sets were enriched by the two cell types; specifically, 70 of 404 gene sets were upregulated by HPGF-C18-cultured BMSCs, while 12 gene sets were significantly enriched by FBS-cultured BMSC (FDR < 25%, Table 3). Interestingly, the gene sets enriched by HPGF-C18 were more likely to include cell proliferation and cell cycle related pathways. These results were consistent with our observed proliferation kinetics. A number of metabolic processing and biosynthetic pathways were also enriched by HPGF-C18 such as Cholesterol homeostasis and Lipid Biosynthetic process. Several signaling pathways such as MTORC1 Signaling and Neurotrophin Signaling pathways were also enriched by HPGF-C18 (Table 3). Moreover, a few immune response gene sets were enriched in HPGF-C18-cultured BMSCs; for example, Interferon-Gamma Response was positively correlated with HPGF-18 culture (Fig. 4b). Among the genes that contributed most to the enrichment of the Interferon-Gamma Response pathway were: CCL2, TNFAIP6, TNFSF10, NFKBIA and FAS suggesting that the immune modulatory functions of HPGF-C18 cultured BMSCs may different from those cultured with FBS. The gene sets enriched in FBS-cultured BMSC included canonical BMSC related signaling pathways, such as Activation of MAPK activity and TGF-beta signaling pathway as well as cell adhesion and ECM pathways including Cell Adhesion Molecules—CAMs and ECM-Receptor Interaction. This implies that lower expression of cell adhesion molecules by HPGF-C18-cultured BMSCs may account for the abnormal “clumping” phenomena. Another interesting gene set enriched by FBS was Epithelial-Mesenchymal transition; the genes contributing most to the enrichment of this pathway included CXCL1, SPARC, IL6, SFRP1, FGF2, TGFBI and DKK1 (Fig. 4c), all of which are representative markers of BMSCs cultured in FBS. Clearly, the results of GSEA analysis indicated that the BMSCs cultured with HPGF-C18 and FBS have different biological activities.

**Table 3:** Gene set enrichment analysis (GSEA) on the global genes

NAME	SIZE	ES	NES	FDR
Enriched gene sets in HPGF-C18 group (FDR < 0.25)				
HALLMARK_CHOLESTEROL_HOMEOSTASIS	24	0.87	2.21	0.00
HALLMARK_MTORC1_SIGNALING	59	0.60	1.99	0.02
HALLMARK_ANDROGEN_RESPONSE	20	0.56	1.87	0.02
KEGG_NEUROTROPHIN_SIGNALING_PATHWAY	26	0.53	1.86	0.02
HALLMARK_MITOTIC_SPINDLE	49	0.71	1.88	0.02
RESPONSE_TO_DNA_DAMAGE_STIMULUS	38	0.69	1.89	0.02
KEGG_P53_SIGNALING_PATHWAY	20	0.69	1.89	0.02
LIPID_BIOSYNTHETIC_PROCESS	32	0.52	1.90	0.03
NUCLEOCYTOPLASMIC_TRANSPORT	20	0.69	1.83	0.03
KEGG_PROGESTERONE_MEDIATED_OOCYTE_MATURATION	25	0.72	1.91	0.03
HALLMARK_INTERFERON_ALPHA_RESPONSE	31	0.59	1.81	0.03
M_PHASE	38	0.72	1.74	0.04
DNA_METABOLIC_PROCESS	65	0.59	1.75	0.04
CELL_CYCLE_PROCESS	62	0.69	1.75	0.04
HALLMARK_ADIPOGENESIS	39	0.47	1.73	0.04
DNA_REPAIR	26	0.72	1.75	0.04
CELL_CYCLE_GO_0007049	95	0.57	1.77	0.04
HALLMARK_APOPTOSIS	57	0.44	1.73	0.04
DNA_RECOMBINATION	15	0.80	1.75	0.04
CELL_CYCLE_PHASE	56	0.67	1.76	0.04
HALLMARK_INTERFERON_GAMMA_RESPONSE	63	0.48	1.77	0.05
CHROMOSOME_ORGANIZATION_AND_BIOGENESIS	24	0.63	1.78	0.05
NUCLEAR_TRANSPORT	21	0.66	1.77	0.05
RESPONSE_TO_ENDOGENOUS_STIMULUS	50	0.55	1.71	0.05
KEGG_CELL_CYCLE	45	0.73	1.70	0.05
MITOTIC_CELL_CYCLE	50	0.69	1.71	0.05
BIOCARTA_HIVNEF_PATHWAY	17	0.59	1.70	0.05
MICROTUBULE_BASED_PROCESS	25	0.62	1.70	0.05
HALLMARK_DNA_REPAIR	22	0.66	1.69	0.06
CELL_CYCLE_CHECKPOINT_GO_0000075	20	0.74	1.68	0.06
ESTABLISHMENT_OF_CELLULAR_LOCALIZATION	71	0.42	1.66	0.07
KEGG_OOCYTE_MEIOSIS	31	0.56	1.66	0.07
GAMETE_GENERATION	37	0.47	1.66	0.07
PROTEIN_TARGETING	22	0.59	1.64	0.07
ESTABLISHMENT_OF_PROTEIN_LOCALIZATION	40	0.47	1.63	0.07
KEGG_APOPTOSIS	21	0.53	1.64	0.07
HALLMARK_G2M_CHECKPOINT	68	0.78	1.64	0.07
M_PHASE_OF_MITOTIC_CELL_CYCLE	26	0.75	1.64	0.07
MACROMOLECULE_LOCALIZATION	47	0.47	1.64	0.07
ORGANELLE_ORGANIZATION_AND_BIOGENESIS	118	0.41	1.62	0.07
REGULATION_OF_CELL_CYCLE	57	0.53	1.62	0.07
MITOSIS	26	0.75	1.64	0.07
CELLULAR_LOCALIZATION	74	0.41	1.61	0.08
DNA_DEPENDENT_DNA_REPLICATION	15	0.70	1.61	0.08
PROTEIN_TRANSPORT	35	0.47	1.60	0.08
CYTOSKELETON_ORGANIZATION_AND_BIOGENESIS	71	0.40	1.58	0.10
DNA_REPLICATION	26	0.63	1.58	0.10
KEGG_PEROXISOME	21	0.46	1.57	0.10
CELL_MIGRATION	26	0.43	1.56	0.11
HALLMARK_ESTROGEN_RESPONSE_EARLY	63	0.40	1.56	0.11
PROTEIN_LOCALIZATION	42	0.46	1.56	0.11
DETECTION_OF_STIMULUS	19	0.55	1.55	0.11
HALLMARK_IL2_STAT5_SIGNALING	59	0.37	1.54	0.12
INTRACELLULAR_PROTEIN_TRANSPORT	30	0.49	1.53	0.12
INTERPHASE	22	0.60	1.53	0.12
CELL_PROLIFERATION_GO_0008283	185	0.34	1.53	0.12
HALLMARK_E2F_TARGETS	60	0.72	1.52	0.13
INTERPHASE_OF_MITOTIC_CELL_CYCLE	19	0.61	1.51	0.13
SEXUAL_REPRODUCTION	48	0.46	1.51	0.13
ORGAN_MORPHOGENESIS	54	0.37	1.50	0.14
ANATOMICAL_STRUCTURE_MORPHOGENESIS	118	0.33	1.50	0.14
HALLMARK_UV_RESPONSE_UP	49	0.37	1.49	0.15
KEGG_PYRIMIDINE_METABOLISM	22	0.55	1.48	0.16
NUCLEOBASENUCLEOSIDENUCLEOTIDE_AND_NUCLEIC_ACID_METABOLIC_PROCESS	263	0.34	1.48	0.16
RESPONSE_TO_STRESS	140	0.32	1.46	0.17
TRANSLATION	30	0.42	1.46	0.17
HALLMARK_SPERMATOGENESIS	47	0.45	1.45	0.18
ACTIN_CYTOSKELETON_ORGANIZATION_AND_BIOGENESIS	38	0.36	1.45	0.18
KEGG_BASAL_CELL_CARCINOMA	16	0.47	1.41	0.22
HALLMARK_TNFA_SIGNALING_VIA_NFKB	69	0.38	1.39	0.24
Enriched gene sets in FBS group (FDR < 0.25)				
HALLMARK_GLYCOLYSIS	54	0.56	1.97	0.02
ACTIVATION_OF_MAPK_ACTIVITY	15	0.65	1.73	0.14
POSITIVE_REGULATION_OF_MAP_KINASE_ACTIVITY	17	0.63	1.72	0.14
KEGG_CELL_ADHESION_MOLECULES_CAMS	48	0.49	1.68	0.15
KEGG_ECM_RECEPTOR_INTERACTION	33	0.54	1.74	0.15
KEGG_FOCAL_ADHESION	61	0.43	1.66	0.16
HALLMARK_HYPOXIA	66	0.51	1.68	0.16
KEGG_TGF_BETA_SIGNALING_PATHWAY	25	0.55	1.68	0.17
EXTRACELLULAR_STRUCTURE_ORGANIZATION_AND_BIOGENESIS	16	0.73	1.74	0.18
FEMALE_PREGNANCY	21	0.59	1.75	0.23
HALLMARK_EPITHELIAL_MESENCHYMAL_TRANSITION	79	0.51	1.78	0.23
TRANSMEMBRANE_RECEPTOR_PROTEIN_SERINE_THREONINE_KINASE_SIGNALING_PATHWAY	18	0.54	1.60	0.25

*ES* enrichment score, *NES* normalized enrichment score, *FDR* false discovery rate

### RT-qPCR Analysis on Differentially Expressed Genes

We also used RT-qPCR to compare the expression of 82 genes among HPGF-C18- and FBS-cultured BMSCs; and the expression 52 genes differed significantly (p < 0.05). Among these genes 22 were up-regulated by HPGF-C18 culture while 30 were up-regulated by FBS-culture (Table 4). Consistent with our microarray data, the expression of a few of the BMSC function related genes did not differ significantly, including CXCL12, IGF1, IL6, IL10, RUNX2 and TGFB1. However, the expression of some genes differed by microarray analysis but did not differ significantly by RT-qPCR analysis including EDN1, FGF2, HGF and VEGFA. Interestingly, TNFSF10 which was confirmed to be up-regulated by HPGF-C18 was expressed by MSC spheres when cultured in chemically defined xeno-free media [27]. Another gene, MMP1, that was up-regulated by HPGF-C18 is critical to MSC migration [28]. Among the genes confirmed to be up-regulated by FBS; HOXA3, FOXF2 and BEX2 were transcription factors; PTGS2, PTGES and PTGIS were immune response modulatory genes; DKK1, SFRP1, and BMP6 were involved in Wnt signaling pathway.

**Table 4:** qRT-PCR analysis of differentially expressed genes

Genes	p value	FDR	FS20/PF10 fold change
PDPN	0.00	0.00	− 28.57
TNFSF10	0.00	0.00	− 27.78
TLR5	0.00	0.00	− 13.89
MMP1	0.00	0.00	− 12.66
BMPR1B	0.00	0.00	− 12.05
TP63	0.02	0.04	− 11.11
CTSC	0.00	0.00	− 9.09
INSIG1	0.00	0.00	− 8.33
CTSK	0.00	0.00	− 8.33
EGR2	0.00	0.00	− 7.69
KLF8	0.00	0.00	− 6.25
PLXDC1	0.00	0.01	− 5.56
COMP	0.00	0.01	− 5.56
IL16	0.01	0.03	− 5.56
ROBO4	0.01	0.03	− 5.26
TGFB3	0.00	0.01	− 4.55
IL27RA	0.00	0.01	− 4.35
CHRD	0.01	0.02	− 3.70
WNT7B	0.02	0.04	− 3.70
MMP13	0.06	0.10	− 3.03
TNFAIP6	0.02	0.04	− 2.63
SMAD1	0.04	0.08	− 2.44
SRGN	0.04	0.08	2.13
TGFBI	0.05	0.09	2.32
IL6	0.04	0.08	2.54
HMOX1	0.05	0.09	2.62
CD40	0.03	0.06	2.88
LTBP1	0.04	0.08	3.36
HOXA3	0.01	0.03	3.53
ALDH1A3	0.02	0.04	3.56
SMAD6	0.03	0.06	3.72
STC2	0.01	0.03	3.74
ID2	0.00	0.01	3.86
MFGE8	0.01	0.02	3.94
CCL26	0.00	0.01	4.32
DKK1	0.00	0.01	4.51
HAS2	0.03	0.05	5.48
PTGS2	0.00	0.00	5.66
ID4	0.00	0.00	6.63
BMP6	0.00	0.00	6.96
TGFB2	0.00	0.00	7.21
KRT18	0.00	0.00	7.96
IL1B	0.00	0.01	8.44
SFRP1	0.00	0.00	8.64
LEPR	0.00	0.00	14.92
PGF	0.00	0.00	19.59
SERPINE2	0.00	0.01	20.77
HAS3	0.00	0.01	21.80
SERPING1	0.00	0.01	32.69
PTGES	0.00	0.00	42.11
PTGIS	0.00	0.00	53.63
BEX2	0.00	0.00	53.81

### Expression of MicroRNAs

We also evaluated the expression of microRNAs using RT-PCR assays. After Quality Control, 256 microRNAs passed the selection criteria and were used for further analysis. Unsupervised clustering using the 256 microRNAs clearly separated all the BMSCs into 2 clusters, one with all the BMSCs cultured with FBS and the other with BMSCs cultured in HPGF-C18 (Fig. 4d), again suggesting that the BMSCs cultured with two media supplements have distinct biological properties. A total of 44 microRNAs were significantly changed (p < 0.05). Among these 44 microRNAs 22 were up-regulated by HPGF-C18 and 22 were up-regulated by FBS (Table 5). Interestingly, microRNAs with the highest changes were related to the osteogenesis of BMSC, including has-miR-146a, has-miR-135b, miR-378, miR-335-5p and miR-210. Hsa-miR-146a regulates the expression of JMJD3 and RUNX2 and thus affects osteogenic differentiation [29]. Hsa-miR-135b is abnormally up-regulated in MSCs from multiple myeloma patients, and it negatively regulates MSCs osteogenesis [30] and the over-expression of hsa-miR-135b results in decreased mineralization [31]. Over-expression of miR-378 attenuates high glucose-suppressed osteogenic differentiation through the targeting CASP3 and activating the PI3 K/Akt pathway [32]. The functional roles of miR-335-5p are, however, controversial. For example, it may activate Wnt signaling and promote osteogenic differentiation by downregulating DKK1 [33], but its over-expression may inhibit the osteogenic and adipogeneic potential of MSC. In addition, miR-335 may also directly target RUNX2 [34] or increase the expression of chondrogenic marker genes [35]. Over-expression of miR-210 significantly reduces MSC apoptosis under oxidative stress, increases cell viability and superoxide dismutase activity [36].

**Table 5:** microRNA identified by RT-qPCR

miRNA	p value	FDR	FS20/PF10 fold change
hsa-miR-146a	0.00	0.07	− 7.69
hsa-miR-135b	0.00	0.07	− 7.69
hsa-miR-378	0.00	0.07	− 4.35
hsa-miR-146b-5p	0.00	0.07	− 4.00
hsa-miR-33a	0.02	0.15	− 3.57
hsa-miR-126*	0.02	0.15	− 3.13
hsa-miR-218	0.01	0.13	− 3.03
hsa-miR-135a	0.00	0.07	− 2.78
hsa-miR-542-5p	0.01	0.09	− 2.50
hsa-miR-15b*	0.01	0.09	− 2.00
hsa-miR-142-3p	0.00	0.07	− 1.96
hsa-miR-450a	0.01	0.11	− 1.89
hsa-miR-29b	0.00	0.07	− 1.79
hsa-miR-424	0.00	0.07	− 1.67
hsa-miR-149	0.01	0.13	− 1.64
hsa-miR-132	0.03	0.17	− 1.61
hsa-miR-20a	0.02	0.14	− 1.59
hsa-miR-455-5p	0.01	0.13	− 1.43
hsa-miR-484	0.01	0.08	− 1.41
hsa-miR-576-5p	0.02	0.14	− 1.33
hsa-miR-15a	0.03	0.18	− 1.18
hsa-miR-28-5p	0.00	0.07	1.19
hsa-let-7i*	0.04	0.24	1.69
hsa-miR-493	0.00	0.07	1.76
hsa-miR-485-3p	0.02	0.15	1.77
hsa-let-7i	0.02	0.15	1.80
hsa-miR-380	0.01	0.14	1.82
hsa-miR-337-3p	0.01	0.11	1.83
hsa-miR-127-3p	0.01	0.09	1.86
hsa-miR-495	0.03	0.17	1.90
hsa-miR-299-3p	0.00	0.07	2.04
hsa-miR-431	0.02	0.15	2.04
hsa-miR-411	0.00	0.07	2.09
hsa-miR-1	0.02	0.14	2.14
hsa-miR-487a	0.01	0.13	2.21
hsa-miR-376c	0.02	0.14	2.21
hsa-miR-487b	0.00	0.07	2.23
hsa-miR-329	0.01	0.14	2.30
hsa-miR-127-5p	0.00	0.07	2.62
hsa-miR-379	0.04	0.24	2.76
hsa-miR-210	0.02	0.14	3.04
hsa-miR-432	0.02	0.15	3.46
hsa-miR-452	0.03	0.21	3.79
hsa-miR-335	0.00	0.07	12.76

Asterick (*) is a part of the microRNA name, indicating the microRNA arises from the 3′ arm of a hairpin

## Discussion

In this study we compared BMSCs cultured from marrow aspirates in media supplemented with HPGF-C18 and FBS. Using the same methods that we used to produce clinical grade BMSCs we found that HPGF-C18 supported the growth of BMSCs from marrow aspirates. BMSC proliferation, CFE, cell surface marker expression and immunosuppression activities were similar among BMSCs grown in HPGF-C18 and FBS. These results are similar to another study which found that HPGF-C18 supported the growth of adipose tissue derived MSCs and surface makers expression was similar for adipose tissue-derived MSC cultured in HPGF-C18 and FBS [23].

BMSCs cultured in HPGF-C18 and FBS were, however, not identical. Differences were found in gene expression profiles, microRNA profiles and cytokines/growth factor concentrations. The most striking differences in gene expression were in pathways involved with cell proliferation, metabolic pathway, immune response, MAPK signaling pathway, TGF-β signaling pathway and cell adhesion pathway. Since many clinical applications of BMSCs involve immune modulation, we evaluated the immune modulatory functions of BMSCs cultured in HPGF-C18 and FBS. We found that BMSCs grown in HPGF-C18 and FBS suppressed lymphocyte proliferation to a similar extent. We then assessed the expression of genes that are involved in the immune modulation using RT-qPCR and found that the expression of many BMSC function related genes did not differ significantly including CXCL12 (SDF1), IGF1, IL6, IL10, RUNX2 and TGFB1. However, the culture supernatant protein levels of SDF1, IL6, TGFB1 and VEGFA differed significantly among HPGF-C18- and FBS-cultured BMSCs. This discrepancy may be due to the post-transcriptional regulation of these genes or technical differences in the two platforms that were used for the analysis. While our results indicate that there are functional differences between BMSCs cultured in HPGF-C18 and FBS, it is not certain if these differences are clinically relevant. Further comparison studies that make use of application specific function assays and models that reflect BMSC potency should be performed prior to substituting HPGF-C18 for FBS for the manufacture of clinical BMSC products.

While the proliferation of BMSCs was similar when grown in media supplemented with 10% HPGF and 20% FBS, we noticed that the BMSCs cultured with HPGF-C18 displayed different morphologies and formed abnormal colonies. The growth of BMSCs in FBS resulted in uniform colonies and monolayer, while growth in HPGF-C18 resulted in some areas where no cells grew and other areas where cells grew in multiple layers. The HPGF-C18 contained some particles and we hypothesized that the irregularities might be due partially to cell attachment to these particles. For the last 2 marrow aspirates we allowed HPGF-C18 to settle and used the supernatant for making cell culture medium. This removed some but not all of the particles. While this reduced the number of areas of irregular growth, they were not completely eliminated and it is possible that complete removal of particles by filtration may further improve BMSC growth. Other mechanisms may also account for the irregularities, differences in BMSC adhesion and mobility may have caused or contributed to the abnormal BMSC growth, for example, HPGF-C18 cultured BMSCs expressed lower levels of cell adhesion molecules such as LAMA3, COL4A4, ITGA8, COL4A2, LAMB2, COL11A2, THBS3, LAMC1 and ITGA8; and expressed high level of motility related gene MMP1. Therefore, it will be interesting to investigate the cell adhesion and motility regulation activities of BMSCs cultured in HPGF-C18 and FBS.

We also noticed differences in the expression of genes belonging to metabolism pathways, and this may be due to differences in the concentrations of growth factors in the two media supplements. Compared to FBS, HPGF-C18 has greater concentrations of Leptin, adiponectin, and immune related proteins, such as EGF, MIP-1, PDGF-BB and CCL1 [23].

Platelet growth factor preparations provide an alternative source of the limited reagent FBS since the growth of cellular therapies will soon result in a shortage of FBS [37]. Besides, there are some advantages in regard to the use of HPGF-C18 rather than FBS; the major advantage is that the use of platelet factor preparations avoids exposure of the recipient of the BMSC products to xenogenic proteins and xenogenic infection.

There are some important differences between HPGF-C18 and other media supplements made from platelets. A relatively large number of apheresis platelet components are used to manufacture each lot of HPGF, approximately 40–50; more than can be easily made in-house by cell processing facilities located in academic health care centers. The use of this relatively large number of apheresis components ensures that the variability among lots is kept to a minimum. In addition, the solvent-detergent treatment used in the manufacture of HPGF-C18 inactivates pathogens. The apheresis platelets used to make platelet-derived media supplements are collected from healthy donors who meet all blood donor healthy history screening and transfusion transmitted disease marker criteria and consequently the possibility that they would transmit a pathogen is low. Solvent-detergent treatment of the pooled apheresis platelets reduces the risk of transmission of a lipid-enveloped virus and inactivates some bacteria. In addition, the final HPGF-C18 product is passed through a sterilizing filter [21], all these measures were taken to make HPGF-C18 a safe alternative to FBS. There may be also functional differences between BMSCs cultured in media supplemented with HPGF-C18 and other platelet-derived media supplements. The quantities of factors released from platelets by freeze/thaw and other methods used to lyse platelets may differ from those released by solvent-detergent treatment. In addition, the process of treating pooled platelet products with solvent-detergent and removing the solvent-detergent by oil extraction and chromatography can also result in the loss of some factors. For example the recovery of TGF-b1, EGF and IGF is over 90% during the hydrophobic interactive chromatography, but the recovery of PDGF-AA, PDGF-AB and PDGF and VEGF was less than 40% [38].

## Conclusions

We found that BMSCs from marrow aspirates could be grown in HPGF-C18 supplemented culture medium; when BMSCs cultured in HPGF-C18 were compared to those cultured in FBS using traditional assays such as proliferation, surface marker expression and inhibition of MLRs, the two types of BMSCs appeared very similar. However, a more comprehensive analysis revealed significant differences at the gene and microRNA expression level and in the composition of the cell supernatants. If HPGF-C18 is to be used in place of FBS for BMSC culture, comparison of BMSCs grown in the two types of media supplements using application specific functional assays that reflect BMSC potency will be necessary.

## Abbreviations

BMSCs: bone marrow stromal cells
FBS: fetal bovine serum
CFE: colony forming efficiency
MLR: mixed lymphocyte culture
GSEA: gene set enrichment analysis
GVHD: graft versus host disease
HPGF-C18: human platelet growth factor C-18
GMP: good manufacturing practices
SOP: standard operating procedure
PDs: population doublings
PCA: principal component analysis
